# Adaptive thresholding for reliable topological inference in single subject fMRI analysis

**DOI:** 10.3389/fnhum.2012.00245

**Published:** 2012-08-25

**Authors:** Krzysztof J. Gorgolewski, Amos J. Storkey, Mark E. Bastin, Cyril R. Pernet

**Affiliations:** ^1^School of Informatics, Nauroinformatics and Computational Neuroscience Doctoral Training Centre, University of EdinburghEdinburgh, UK; ^2^Brain Research Imaging Centre, a SINAPSE Collaboration Centre, University of EdinburghEdinburgh, UK; ^3^School of Informatics, Institute for Adaptive and Neural Computation, University of EdinburghEdinburgh, UK; ^4^Health Sciences (Medical Physics), University of EdinburghEdinburgh, UK

**Keywords:** mixture models, random field theory, false negative errors, spatial accuracy, reliability

## Abstract

Single subject fMRI has proved to be a useful tool for mapping functional areas in clinical procedures such as tumor resection. Using fMRI data, clinicians assess the risk, plan and execute such procedures based on thresholded statistical maps. However, because current thresholding methods were developed mainly in the context of cognitive neuroscience group studies, most single subject fMRI maps are thresholded manually to satisfy specific criteria related to single subject analyzes. Here, we propose a new adaptive thresholding method which combines Gamma-Gaussian mixture modeling with topological thresholding to improve cluster delineation. In a series of simulations we show that by adapting to the signal and noise properties, the new method performs well in terms of total number of errors but also in terms of the trade-off between false negative and positive cluster error rates. Similarly, simulations show that adaptive thresholding performs better than fixed thresholding in terms of over and underestimation of the true activation border (i.e., higher spatial accuracy). Finally, through simulations and a motor test–retest study on 10 volunteer subjects, we show that adaptive thresholding improves reliability, mainly by accounting for the global signal variance. This in turn increases the likelihood that the true activation pattern can be determined offering an automatic yet flexible way to threshold single subject fMRI maps.

## Introduction

The final outcome from fMRI analyzes is a map showing which areas are most likely involved in certain sensory-motor or cognitive skills. After appropriate data pre-processing, a general linear model (GLM) is fitted to the measured signal and a *T*-test looking for difference between conditions or between a given condition vs. rest is performed. The result is a 3D volume of *T*-values. Given these *T*-values, each voxel is labeled as being “active” (involved in the task) or “not-active” (not involved in the task) based on an *ad-hoc* threshold. This procedure has been successfully used in the context of cognitive neuroscience group studies for population inference. However, three major problems need to be addressed in order to improve inference at the subject level when used for clinical decision making, namely: (1) the impact of signal-to-noise ratio (SNR) on thresholding, (2) the relative importance of Type I versus Type II error rates, and (3) the spatial accuracy of the thresholded maps. In this paper we investigate how these issues affect statistical maps and describe a new adaptive thresholding method which improves cluster detection and delineation.

SNR is usually higher in group studies than in single subject fMRI. In group studies, one averages the effect (beta parameters of the GLM) observed in multiple subjects, which usually leads to a stronger signal than that obtained for just one subject. In addition, statistical significance is assessed in comparison to the between subject variance, which is less dependent on scanner related noise than within subject variance. In single subject analyzes, the effects are usually estimated on a single set of scans with comparison to the between scan variance. In this context, the SNR can be low due to scanner noise with potentially high between scan variance. This is particularly true in the clinical context in which patients are often advanced in age or impaired by medical conditions (Stippich et al., 2007), resulting in reduced scanning time (less signal) or increased motion (more noise). In consequence, researchers often threshold single subject maps manually based on prior anatomo-functional knowledge and expectations (O'Donnell et al., 2011) rather than using the signal properties or the statistical values. Such a liberal approach is problematic as it may prevent reliable results. Depending on the researcher, clinician, or radiologist, different thresholds will be used leading to different inferences. Single subject fMRI analyzes thus require a thresholding method that gives more reliable results.

Cognitive neuroscience group studies have focused on avoiding false positives, whereas in the clinical context, false negatives are also an issue. The biggest concern of the researcher or clinician using fMRI is validity, i.e., is the brain activation that is observed real or an artefact? Statistical methods reflect this point of view by controling for the probability of a false positive error, i.e., reporting an activation that is not present. By contrast, the goal of a surgical procedure such as tumor resection is to remove as much diseased tissue as possible while preserving mental and cognitive capabilities. In this context, surgeons are not only interested in delineating eloquent cortical areas, but also in delineating the tissue that is not involved with a particular cognitive skill. Therefore, the error of reporting an area as not active, and safe to cut out, when in fact it is active (a false negative error) has more profound consequences than a false positive error. In single subject fMRI which is used for clinical decision making, it is thus more important to have a method that provides a good balance between the two expected error rates rather than one that controls perfectly for only one of the two error rates.

The spatial extent of active areas is also of greater importance in the clinical context than in cognitive neuroscience studies. In the latter, it is often sufficient to answer the question of where certain neuronal processes take place in an average brain. As a consequence, many publications report only the peak coordinates of activation. However, in the clinical context, the precise location matters. In the case of presurgical planning for example, decisions about the safety of the procedure and extent of the resection are made based on the distance between a tumor and the eloquent cortex as revealed by fMRI. The statistical threshold used influences this distance by changing the spatial extent of activated areas, whilst it usually doesn't impact on the peak location. Therefore, the thresholding method used in single subject analyzes must allow a good delineation of the true underlying signal extent.

From a logical perspective, since in most paradigms used it is expected that some signal is present in the brain, it seems reasonable to analyze the data assuming a signal model (Turkheimer et al., 2004). Mixture models represent the entire distribution of statistical values for a given space as a mixture of the “active” and “noise” distributions. The first application of mixture models to fMRI data was proposed by Everitt and Bullmore (1999). In this initial work, voxels values of a statistical parametric map (SPM) were modeled as a mixture of central and non-central chi-square distributions thus producing a distribution corresponding to no activation, and another distribution corresponding to the presence of some (either positive or negative) activation. After fitting the model, posterior probabilities were obtained for each voxel to be active or inactive and this SPM was thresholded to reveal significantly activated areas. At the heart of such approach is the assumption that signal and noise, in particular the null distribution, can be separated via modeling. This idea was later adopted by others (Hartvig and Jensen, 2000; Woolrich et al., 2005) who incorporated spatial priors to account for the correlation between voxels. Both Hartvig and Jensen (2000) and Woolrich et al. (2005) used Markow random fields (MRF) to spatially reguralize labeling of the statistiacal map, although Woolrich et al. (2005) were the first to train parameters of the MRF from the data in a Bayesian way. More recently, Pendse et al. (2009) considered a mixture of Gaussians to model the null distribution in an attempt to improve voxelwise false discovery rate (FDR). They have used Bayesian information criterion (BIC) to choose how many mixture components are required accurately describe the data. However, in this method the inference was carried out on the voxel level without taking into account spatial characteristics of the signal such as cluster size and suffers from problems with interpreting which Gaussians correspond to either noise or activations class.

The aim of our approach is to perform inference on the cluster level and at the same time provide a good balance between false positive and negative errors in the delineation of activation borders. We therefore propose a Gamma-Gaussian mixture model as a method to account for distributions of *T*-values in SPMs (Woolrich et al., 2005) and set a threshold specific to the data at hand. A natural way to determine this threshold is to take the point that separates signal from noise. This point is the crossing between the Gaussian, the model corresponding to no activation, and the Gamma distribution, the model corresponding to positive activations, and provides a good trade-off between false positive and negative (voxel-wise) rates. Finally, once this threshold is established, topological inference via FDR correction over clusters (Chumbley and Friston, 2009) is used to correct for the number of tests performed while accounting for spatial dependencies across voxels, thereby explicitly controling for Type I cluster rate. This heuristic approach combines advantages of the different methods mentioned above. Specifically it relies on a simple model of the SPM, allows adaptive thresholding, and accounts for multiple comparisons in the context of topological inference.

## Materials and methods

### Gamma-gaussian mixture model

Following Woolrich et al. (2005), the *T*-value distribution from a SPM covering all brain voxels is modeled using a Gamma-Gaussian mixture model, with the Gaussian distribution as a model for the null distribution (no activation) and Gamma distributions as models for the negative (deactivation) and positive (activation) distributions. Note that due to high degrees of freedom in a typical fMRI experiment, i.e., the number of time points greatly exceeds number of regressors, a normal distribution is good approximation of Student's *t*-distribution. In practice, three different models are fitted to the data, namely:

$$\begin{array}{l} p(x)=N(x|\mu ,\;\sigma )\\ p(x)=\pi_{N}N(x|\mu ,\;\sigma )+\pi_{A}\text{Gamma}(x+\mu |k,\;\theta )\\ p(x)=\pi_{D}\text{Gamma}(-(x+\mu )|k_{D},\;\theta_{D})+\pi_{N}N(x|\mu ,\;\sigma )+\pi_{A}\text{Gamma}(x+\mu |k_{A},\;\theta_{A}). \end{array}$$

with *x* representing all the *T*-values, *p(x)* the probability distribution, μ is the mean and σ the standard deviation of the Gaussian (N) component, *k* is the shape parameter and θ the scale parameter of the Gamma component(s), and π is the proportion/contribution of each component (*N* for Gaussian/noise, *A* and *D* for Gamma/activation–deactivation).

Model 1 is fitted using maximum likelihood estimator, and Models 2 and 3 are fitted using an expectation-maximization algorithm (Dempster et al., 1977). In all three models, the Gaussian component represents the noise. In Model 2, the Gamma component corresponds to the activations. In Model 3, Gamma corresponds to the activation and deactivation classes. Note that Gamma components are shifted by the estimated mean of the noise (Gaussian) component (the non-spatial model described in Woolrich et al., 2005 did not incorporate such shift). The Gaussian distribution is a natural choice to model noise, while the Gamma distributions have the advantage of being restricted to cover only values above (activation) or below (deactivation) the Gaussian mean. This helps to force these components to fit the tails of the distribution. For each model, BIC is calculated and the model with the highest score is selected. Although only Model 2 can be used to fit the data as some signal is expected, fitting all three models offers much more flexibility. In particular, compared to other approaches (e.g., Pendse et al., 2009), the explicit model selection via BIC allows the case when no signal is present (Model 1) to be determined, and avoids having to attribute subjectively model components to noise or (de)activations, i.e., Models 2 and 3. Similarly, in the case that deactivations are present, the mean of the noise component in Model 2 is biased because the left tail is not well estimated and so is the positive Gamma component; having an explicit model for this case (Model 3) allows for deactivations to be present without interfering with the threshold. In the case that Models 2 or 3 are selected, each voxel is assigned a label (activation, deactivation, and noise) corresponding to the component with the highest posterior probability. In these cases, the highest *T*-value among voxels belonging to the noise class is chosen as the new cluster forming threshold.

### Thresholding procedure

**Figure 1:**
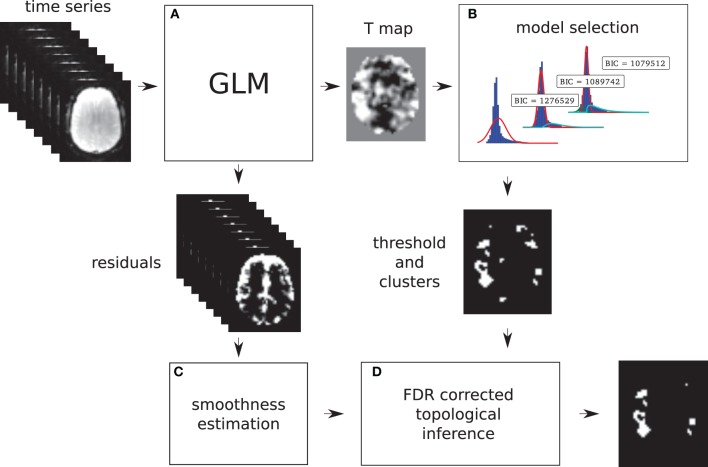
**Overview of the topological FDR inference using our Gamma-Gaussian mixture model to set adaptively the cluster forming threshold**. GLM produces T-map and residuals **(A)** Three models are being fitted to the voxels from the T-map **(B)** Models include a combination of deactivation (green), noise (red), and activation (cyan) components. Smoothness of the image is estimated from the residuals **(C)** Threshold estimated from the winning model **(B)** and smoothness of the image are used to perform topological inference on cluster extent **(D)**.

Models 2 and 3 allow a probability of being active to be assigned to every voxel. This probability is used to find a threshold that corresponds to a point in which the probabilities of positive Gamma and Gaussian are equal, i.e., the crossing point between the two distributions. This equal probability threshold thus separates signal from noise. At this stage, topological FDR is used to control for false positive clusters (Chumbley and Friston, 2009). In the situation when Model 2 or 3 is selected in the first stage, thus providing evidence of true activation, but none of the clusters survive the topological FDR step, a heuristic threshold is applied to make sure that some activation is found. In this case, the cluster with the highest sum of *T*-values is labeled as active. We have found that this situation can arise in a few clinical cases, and this heuristic approach solves the issue. An overview of the method can be found in Figure 1. Freely available implementation of the method is available at https://github.com/chrisfilo/Adaptive-Thresholding for both Nipype (python) and SPM8 (Matlab®).

### Simulations

To investigate the performance of each method, a total of 2500 time series were simulated. Each simulated time series included eighty planes of 128 × 128 elements. Half of the planes included just normally distributed noise (μ = 0, σ^2^ = 1) and the second half included a pattern of activation added to the noise. The pattern consisted of six squares of different sizes (4 × 4, 8 × 8, 12 × 12, 16 × 16, 20 × 20, and 24 × 24). Because temporal aspects of the fMRI signal such as autocorrelation were not the focus of this research, the time series consisted of only two blocks, namely 40 planes of “rest” followed by 40 planes of “task”. All of the planes were convolved with a Gaussian filter of full width half maximum (FWHM) of 6 mm. The height of the pattern, representing the strength of the signal, was also varied (0.04, 0.08, 0.16, 0.32, and 0.64) and for each of the five signal strengths, data (signal + noise) were simulated 500 times.

Time series generated in this way were fitted with a GLM model with a single regressor, and no autoregression, high-pass filtering, or convolution with a hemodynamic response function. Because neither the simulated signal nor the fitted model included any temporal dependencies, the selected design (40 “rest” followed by 40 “task” planes) was no different from any other combination, e.g., 5 “rest” followed by 5 “task” blocks repeated eight times. A single contrast was estimated and thresholded using topological FDR with three different cluster forming thresholds. Two fixed cluster forming thresholds were used across all 2500 SPMs, specifically a *p*-values of 0.05 with family wise error (FWE) correction (*T*-value of 4.47) and 0.001 uncorrected (*T*-value of 3.19). These thresholds were chosen as they correspond to defaults values used in the SPM software package (http://www.fil.ion.ucl.ac.uk/spm/) and we refer to them as **fixed thresholds (FT 0.05 FWE and FT 0.001)**. This contrasts with the cluster forming thresholds obtained with the Gamma-Gaussian mixture model which by nature change with the data. Note that for each map, all three Gamma-Gaussian models were always fitted and the model that best described the data according to our BIC selected to set the cluster forming threshold. In these simulations, Model 2 was always the best model since there was always some signal plus noise, which also showed that the model selection worked. We refer to these thresholds as **adaptive thresholds (AT)**. These simulations therefore allow the performance of AT and FT to be compared in terms of false positive and false negative cluster rates, spatial accuracy, and influence of global signal variation.

#### False positive and negative cluster rates

A false positive cluster was defined as a supra-threshold group of connected voxels that did not overlap to any extent with the squares in the true activation pattern. By analogy, a false negative cluster should be an infra-threshold group of connected voxels corresponding to a true activation pattern. Because one cannot obtain negative clusters, we simply defined the false negative cluster rate as the rate of true patterns that were not detected, i.e., missed. Comparison of AT with FT were performed in a pair-wise fashion for every simulated time series. First, false positive and negative cluster rates were calculated for all three thresholding methods. Second, the differences (trade-off) between false positive and negative rates were computed. Third, difference between AT and the two default FT values (0.001 uncorrected and 005 FWE corrected) for the absolute value of the trade-offs were obtained. Finally, a percentile bootstrap, resampled with replacement of the differences between thresholding methods, was used to estimate *p*-values and confidence intervals of the mean differences and multiple tests correction was applied using the Benjamin-Hochberg (B-H) method maintaining FDR at the 0.05 level (Benjamini and Hochberg, 1995). Computing the difference between false positive and negative rates allowed testing for the average improvement of AT over the two default FT values in terms of trade-off, i.e., values around 0 mean a good balance between the two types of error. However, if the method gives two very large errors it can still give a good trade-off. We thus also computed the total sum of type I and type II errors, ensuring that AT doesn't lead to overall larger errors.

#### Spatial accuracy

**Figure 2:**
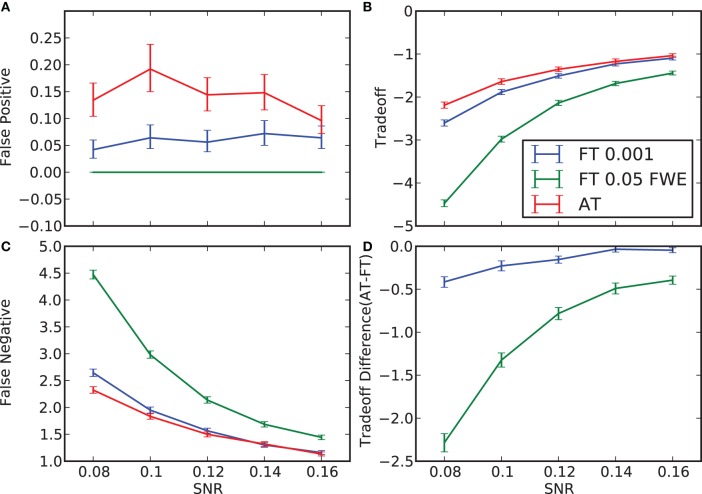
**False positive and negative cluster rates**. On the left are displayed the mean false positive **(A)** and negative **(C)** cluster rates. On the right are displayed the mean clusters trade-off **(B)** and the difference between AT and the two default FT values for this trade-off **(D)**. Whiskers represent 95% confidence intervals estimated using a percentile bootstrap for each SNR independently (uncorrected for multiple comparisons).

Spatial accuracy was defined as the difference between the overestimation and underestimation of cluster's borders, i.e., it reflects if cluster's borders were well delineated. For a given true cluster, the degree of underestimation was defined as the number of voxels that were falsely declared as not active, and the degree of overestimation was defined as the number of voxels that were falsely declared as active. Using these definitions, cluster borders can be simultaneously overestimated (voxels declared active that should not be) and underestimated (voxels declared non-active that should not be—see Figure 2). Note that only true positive clusters that were observed in all thresholding methods were used for this analysis to make the count fair between the three thresholds. In addition, each cluster size was analyzed separately. Comparisons between AT and FT were performed in a pair-wise manner using a percentile bootstrap on the Harrell–Davies estimates of the median differences. Multiple tests correction was applied using B-H method maintaining FDR at the 0.05 level.

#### Influence of global effects

One major confound that can influence thresholding results is a global (occurring in all voxels) signal change that is correlated with the stimuli. This has been commonly referred to in the literature as “global effect” (Friston et al., 1990; Aguirre et al., 1998; Gavrilescu et al., 2002; Junghöfer et al., 2005; Murphy et al., 2009). This global effect results in a shift of all *T*-values by a constant. We simulated this effect by taking all the *T*-maps of signal height 0.08 and adding a random constant (normally distributed, μ = 0, σ^2^ = 1) to all values. *T*-maps created this way were thresholding using AT and the two default FT values. Here only simulations with low SNR were manipulated to investigate the noisiest scenario. Dice coefficients (Dice, 1945) were computed for every simulation between the thresholded shifted and unshifted maps. This allowed the reliability of thresholding methods to be investigated in the context of global effects. Comparison between AT and FT was performed using a percentile bootstrap of the mean of the pair-wise differences between Dice coefficients.

### fMRI data and reliability analyzes

#### Subjects

Eleven healthy volunteers were recruited. One subject had to be discarded due to problems with executing the task. The remaining 10 subjects (7 left handed) included 4 males and 6 females with a median age at the time of scanning of 52.5 years (range 50–58 years). The study was approved by the local NHS Research Ethics Committee.

#### Paradigm

Subjects had to move a body part corresponding to a picture. The following instructions were used: “You have to tap your index finger when you see a picture of a finger, flex your foot when you see a picture of a foot, and purse your lips when you see a picture of lips”. A block design with 3× 15 s activation periods with 15 s rest periods was used. In every block, subjects moved the index finger of their dominant hand, or flipped their dominant foot or pursed their mouth. Movement was paced (0.4 Hz) by the visual stimuli. Four trials were used for training before data acquisition. Four volumes were acquired for signal stabilization before stimulus presentation. There were five repetitions of each activation blocks for a total scan time of 7 min 40 s. The paradigm was implemented using Presentation® Software (Neuro Behavioural Systems) and stimulus synchronization and presentation was provided via NordicNeuroLab (http://www.nordicneurolab.com/) hardware.

#### MRI parameters

Scanning was performed using a GE Signal HDx 1.5 T clinical scanner at the Brain Research Imaging Centre, University of Edinburgh (http://www.bric.ed.ac.uk/). Each volunteer was scanned twice, two (eight subjects) or three (two subjects) days apart. fMRI data were acquired using echo-planar imaging (EPI) with a TR of 2.5 s, a TE of 50 ms, a flip angle of 90°, 30 slices per volume (4 mm thick) with interleaved acquisition, an acquisition matrix of 64 × 64 with a FOV of 256 × 256 mm (voxel size 4 × 4 × 4 mm). A high resolution T1-weighted coronal volume (156 slices of 1.3 mm thickness, acquisition matrix 256 × 256 and FOV 256 × 256 mm; voxel size 1 × 1 × 1.3 mm) was also acquired on both days.

#### Data analysis

Data were processed using SPM (http://www.fil.ion.ucl.ac.uk/spm/) and FSL (http://www.fmrib.ox.ac.uk/fsl/) tools within the Nipype framework (http://nipy.sourceforge.net/nipype/; Gorgolewski et al., 2011). For every subject, the T1-weighted volumes from both sessions were coregistered, resliced, and averaged. A DARTEL template was created using averages from all subjects (Ashburner, 2007). Additionally a brain mask was estimated from each average using BET (Smith, 2002). For the fMRI data, the first four volumes, during which the scanner reaches steady state, of every EPI sequence were discarded and remaining images were slice time corrected. Finger, foot, and lips sequences of left-handed subjects were flipped along the Z-Y plane. For every subject, all slice time corrected volumes from all tasks and sessions were realigned and resliced to their mean. The mean volumes were coregistered to the subject's average T1-weighted volume and the resulting affine transformation was applied to headers of realigned files. Each EPI volume was then normalized using the DARTEL template and corresponding flow field. Finally, data were smoothed with an isotropic 8 mm FWHM Gaussian kernel. Each session was analyzed separately. GLM (Friston et al., 1994) was used to fit a design matrix consisting of an autoregressive filtering matrix (AR1), a high pass filter (128 Hz), the task parameters (block onsets and duration for each body part), the 6 realignment parameters, and multiple artefacts regressors. High frequency motion or global effect artefacts were obtained using the Artifact detection toolbox (http://www.nitrc.org/projects/artifact_detect/). Only voxels within the previously estimated brain mask were included in model fitting.

The test-retest reliability was defined by the amount of overlap between thresholded maps from the two scanning sessions (Dice coefficient). *T*-value contrasts were computed for each body part and the resulting maps were thresholded with a cluster threshold of 0.05 FDR corrected but using AT and the two default FT values as in the simulations. For every subject, contrast and thresholding method, Dice similarity (overlap) was calculated between the two sessions. This has previously been performed for full brain and within a mask including areas 4a and 4p (Geyer et al., 1996), and is available in the anatomy toolbox (Eickhoff et al., 2005, 2006, 2007). The mask was generated in MNI space and resliced to DARTEL template dimensions. Comparison of thresholding methods was performed using a percentile bootstrap on the differences between Dice coefficients. Finally, to further investigate the impact of AT and FT on reliability, the Dice values were computed using multiple threshold combinations between sessions, and AT and FT located in this space. This allowed an understanding of the underlying behavioral of our reliability metric in relation to different cluster forming thresholds.

## Results

### Simulations

#### False positive and negative cluster rates

In terms of sensitivity or false negative clusters, AT outperformed both default FT values. The difference was largest for lower SNR and a FT of *p* = 0.05 FWE corrected. In the case of FT of 0.001 uncorrected, AT was more sensitive only for SNR values below 0.14 (Table 1 and Figure 2C). This increase in sensitivity for AT, especially at low SNR, also came with a higher number of false positive clusters than FT (see Figure 2A). However, this increase in false positive clusters was comparatively small to the gain in sensitivity such as the total number of errors was similar to FT; in fact even better than FT in most cases (see Table 2). Statistical analysis of the differences between false positive and negative clusters shows that AT has a better trade-off than both default FT values (Table 1), with the biggest advantage for low SNR values. With high SNR, AT and FT (0.001 uncorrected) gives similar results (Figure 2D).

**Table 1:** **Statistical analysis the pair-wise difference (AT-FT) comparison of cluster error trade-off**.

		**SNR**				
		**0.08**	**0.1**	**0.12**	**0.14**	**0.16**
AT–FT 0.001	High CI	−0.350	−0.168	−0.118	0.002	−0.018
	Mean	−0.414	−0.228	−0.154	−0.034	−0.046
	Low CI	−0.480	−0.288	−0.194	−0.070	−0.076
	q-vals	**<0.0001**	**<0.0001**	**<0.0001**	0.059	**0.003**
AT–FT 0.05 FWE	High CI	−2.182	−1.242	−0.712	0.428	−0.346
	Mean	−2.284	−1.324	−0.782	−0.490	−0.394
	Low CI	−2.390	−1.404	−0.856	−0.550	−0.444
	q-vals	**<0.0001**	**<0.0001**	**<0.0001**	**<0.0001**	**<0.0001**

Q-values correspond to p-values corrected for multiple comparisons using Benjamin-Hochberg method for controling FDR.

Statistically significant tests are highlighted in bold.

**Table 2:** **Statistical analysis of the pair-wise difference (AT-FT) comparison of the total number of errors (false positive + false negative)**.

		**SNR**				
		**0.08**	**0.1**	**0.12**	**0.14**	**0.16**
AT–FT 0.001	High CI	−0.168	0.072	0.06	0.132	0.036
	Mean	−0.23	0.012	0.022	0.094	0.006
	Low CI	−0.292	−0.048	−0.018	0.058	−0.024
	q-vals	**<0.0001**	0.772	0.50222222	**<0.0001**	0.772
AT–FT 0.05 FWE	High CI	−1.91	−0.866	−0.424	−0.16	−0.162
	Mean	−2.016	−0.956	−0.494	−0.218	−0.214
	Low CI	−2.126	−1.044	−0.566	−0.276	−0.266
	q-vals	**<0.0001**	**<0.0001**	**<0.0001**	**<0.0001**	**<0.0001**

Q-values correspond to p-values corrected for multiple comparisons using Benjamin-Hochberg method for controling FDR.

Statistically significant tests are highlighted in bold.

#### Spatial accuracy

**Figure 3:**
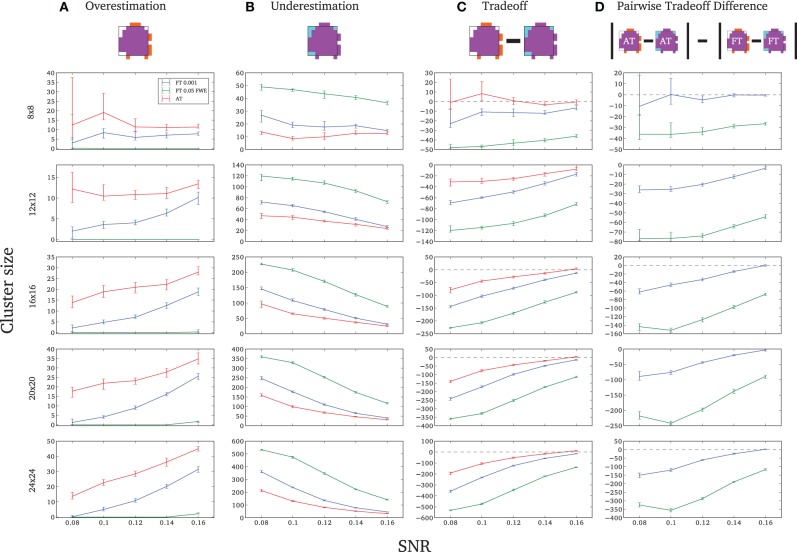
**Illustration of over and underestimation and performances of the different thresholding methods**. At the top is an illustration of an observed cluster (purple) over the true underlying signal (square outline). Estimated voxels outside of the true border are in orange and missed voxels inside the border are in cyan. Below, graphs represent four biases. On the left are displayed the H-D estimates of the median cluster extent overestimation **(A)**, i.e., the number of false positive voxels for a particular cluster. Next are displayed the H-D estimates of the median cluster extent underestimation **(B)**, i.e., the number of false negative voxels for a particular cluster. Next is displayed the overestimation and underestimation trade-off **(C)**, i.e., differences of the H-D estimates of the medians. Finally on the right hand side is displayed the pairwise comparison between AT and FT trade-offs **(D)**. Each row corresponds to different cluster size and whiskers represent 95% confidence intervals. Due to the fact that the smallest cluster (4 × 4) was found by all of the thresholding methods only in a handful of runs it was excluded from this plot.

Due to the fact that the smallest cluster was found by all of the thresholding methods in only a handful of runs it was excluded from further analyzes; in other words there were not enough true positives to reliably estimates border accuracy. For the remaining cluster sizes, AT outperformed both default FT values in terms of underestimation of borders, i.e., it showed fewer false negative voxels (see Figure 3B), but at the same time it performed worst in terms of overestimation with more false positive voxels (see Figure 3A). However, the difference was such that AT had a better overall spatial accuracy, i.e., trade-off between over and underestimation (see Table A1 and Figures 3C,D). AT provided a statistically significant improvement in terms of the border over/under estimation when compared to both of the two FT values. As in the cluster analysis the effect was stronger for lower SNR levels, although in case of the highest tested SNR, 0.16, FT 0.001 performed equally well as AT.

#### Influence of global effects

**Figure 4:**
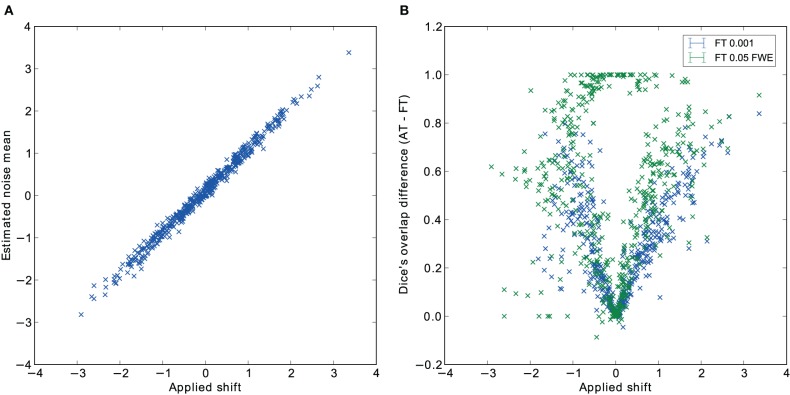
**Estimated mean of the noise component versus the applied distribution shift (A) and the improvement of AT over FT with respect to the applied distribution shift (B)**.

Pair-wise difference between Dice coefficients for AT and FT show an overall higher immunity to global noise for AT than FT (mean difference: 0.32 for FT 0.001 uncorrected; *p* < 0.0001 and 0.51 for FT 0.05 FWE; *p* < 0.0001). Global effects lead to a shift of the overall distribution such that the FT procedures created clusters of different sizes. By contrast, AT was able to recover from this confound by shifting the center of the Gaussian in the mixture model, thus creating clusters of similar sizes. Looking at the correlation between the applied shift and the estimated mean of the Gaussian component (see Figure 4) showed that the Gamma-Gaussian mixture model accurately estimated this effect (*r* = 0.99, *p* < 0.0001). Plotting Dice coefficient differences against the applied distribution shift (see Figure 4) showed that the increase in reliability came from this shift such that it varied proportionally with the absolute value of the applied shift (FT 0.001 uncorrected *r* = 0.69; p < 0.0001 and FT 0.05 FWE *r* = 0.34; *p* < 0.0001). This demonstrates how big an influence global noise can have on the thresholded maps. Due to flexibility in the assumptions of the noise distribution, in that the mean does not necessarily have to be zero, AT managed to accurately estimate the confounding shift. This lead to better recovery of the unshifted maps, which in real world would translate to better reliability for the same subject between two sessions.

### Reliability experiment

**Figure 5:**
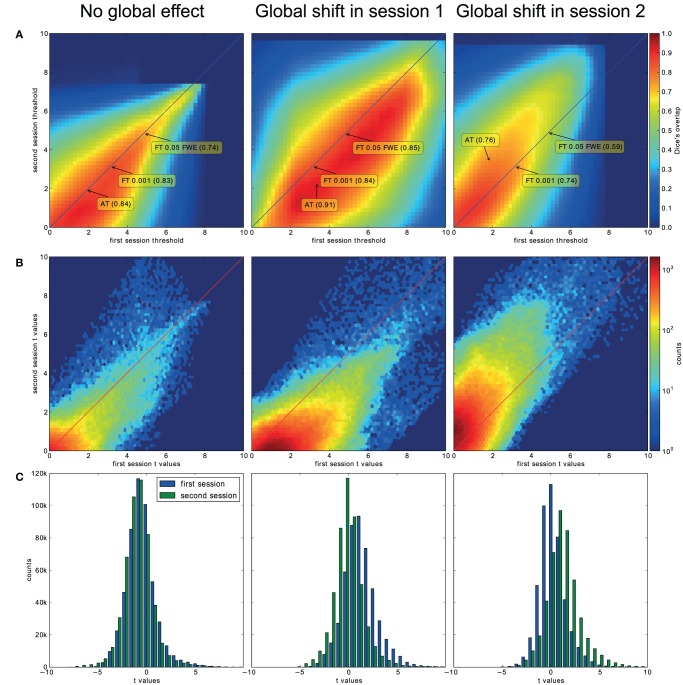
**Analysis of the T-map reliability of three selected subjects**. The top row **(A)** shows between session Dice coefficients for different pairs of cluster forming thresholds. The middle row **(B)** shows the upper right quadrant of the joint distribution of the unthresholded *T*-values, while the bottom row **(C)** shows distributions of *T*-values from the first and the second session. “No global effects” (example from finger contrast for subject (1) illustrates the case where choosing the same threshold for both session is the optimal course of action; the joint distribution confirms this showing lack of a consistent between session value shift, while AT manages to infer this without having access to the joint distribution. “Global shift session 1” (example from lip movement contrast for subject (2) shows a shift of values between the sessions. This is clear not only from the joint distribution but from the two separate distributions. This allows AT to choose a lower threshold for the second session and optimize the Dice coefficient value. “Global shift session 2” (example from foot contrast for subject (3) presents a shift in the opposite direction.

**Figure 6:**
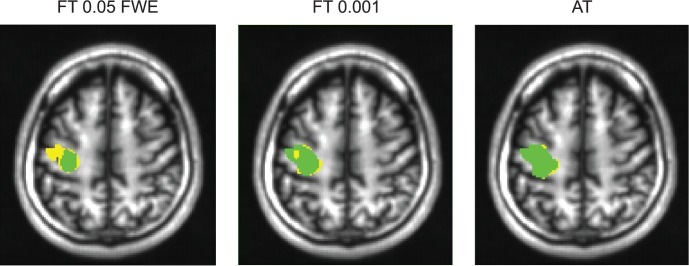
**Thresholded statistical maps of the first subject used in Figure 5**. Voxels in green were labeled active in both sessions. Voxels in yellow were labeled active only in of the sessions (and non active in the other). More yellow voxels indicate bigger between session variance and smaller test–retest reliability.

For the three evaluated contrasts of the motor task (finger, foot, and lips), AT provided improvement in terms of between session Dice overlap over both default FT values (see Table 3). Mapping of the parameter space (see Figures 5A,B) showed that many combinations of thresholds can lead to high Dice overlap, and that highest values were obtained when different thresholds between sessions were used. The reason behind this phenomenon is that maximum *T*-values are often shifted between sessions as evidenced by looking at the joint distribution of *T*-values. Indeed the tail of the joint distribution is off-diagonal (see Figure 5B), meaning that voxels in the second scan session have higher or lower *T*-values than the same voxels in the first session. This effect is mostly observed when there is a shift of the overall distribution, i.e., in the context of a “global effect” (Friston et al., 1990) such as when temporal noise correlates with the stimuli sequence and affects the whole brain. Such a between session shift of *T*-values in a test-retest study has recently been reported by Raemaekers et al. (2012). AT attempts to estimate and correct for this effect by allowing the Gaussian component to have non-zero mean and having the “activation” and “non-activation” components range fixed to that mean, leading to a choice of a pair of thresholds optimal in terms of Dice overlap (see Figure 5C). The effect of thresholding using fixed vs. adaptive methods can be seen on Figure 6.

**Table 3:** **Statistical analysis of the pair-wise comparison of Dice coefficients (AT-FT)**.

	**Full brain**		**ROI**	
	**0.001**	**0.05 FWE**	**0.001**	**0.05 FWE**
High CI	0.057	0.136	0.053	0.243
H-D Median	0.026	0.075	0.021	0.131
Low CI	0.003	0.034	0.001	0.067
*P*-value	**0.026**	**<0.0001**	**0.037**	**<0.0001**

P-values were not corrected for multiple comparisons.

Statistically significant tests are highlighted in bold.

## Discussion

Single subject fMRI analyzes have different requirements than group studies mainly because the SNR is often lower, and one wants to reveal specific or expected areas and delineate their spatial extent. For these reasons, a fixed threshold strategy is rarely adopted and each subject's *T*-value map tends to be thresholded differently. Here, we propose a method that thresholds each subject's statistical map differently, but follows an objective criterion rather than a subjective decision. Indeed, we show that our adaptive thresholding method outperforms default fixed thresholds both in terms of trade-off between Type I and Type II cluster error rates and in terms of spatial accuracy. This increase in spatial accuracy can also be inferred from the reliability results. While validity and reliability can be separated in various conditions, we can infer that, for fMRI, the most valid voxels are the ones detected reliably. Valid and reliable voxels usually correspond to voxels located at the core of a cluster while non-valid and non-reliable voxels are located at the cluster borders. Since AT leads to higher reliability than FT, we can infer that it also improves clusters delineation in real data sets.

A major source of noise in fMRI time series relates to global effects. Because of the shift of the overall *T*-value distribution below or above 0, a fixed threshold strategy can lead to the under or overestimation of the true signal. By contrast, we show that AT can correct for “global effects” by shifting the mean of the Gaussian component in our Gamma-Gaussian mixture model. This ability to adapt to noise translates to improved reliability in a test-retest study on healthy controls. A similar approach has been used before to remove global effect biases in a session variability study by Smith et al. (2005), but not in context of thresholding statistical maps.

Mixture models have been used previously to threshold statistical maps. Most recently Pendse et al. (2009) have used a mixture of Gaussians to improve FDR control by estimating the empirical null. There are two major differences between this and our approach. Firstly, inference is performed on the cluster level as described by Chumbley and Friston (2009), and directly incorporates spatial dependencies between voxels. Secondly, when it comes to border delineation, we are interested in the balance between false positive and negative errors. Controling for voxelwise FDR does not solve the problem of false negative errors, which as we argue above, are very important in the clinical context and for single subject analyzes in general. The closest method to our approach is work presented by Woolrich et al. (2005). Their model also uses a Gamma-Gaussian mixture model, but incorporates spatial information through Markov random field instead of Gaussian Random fields. Such model is harder to fit than the Gaussian Random fields approach due to the problems of finding the right spatial regularization coefficients. Also, both approaches do not assume 0 centrality for the noise component, whereas our model shifts the activation and deactivation Gamma distribution according to the estimated Gaussian (noise) mean, thereby providing immunity to global noise.

In our method we have decided to choose a cluster forming threshold that would minimize the sum of voxelwise false positive and false negative errors. Modeling the *T*-values distributions using a mixture of gamma and Gaussian distributions allows performing such optimization. Higher thresholds yield more false negative errors and lower thresholds yield more false positive errors. However, when it comes to the sum of all errors there is an optimal threshold which is equal to the crossing point between the Gaussian and Gamma distributions. Our simulations confirmed this theoretical relation (see Table 2). There have also been other attempts at creating adaptive thresholding methods. One of the most notable is activity mapping as percentage of local excitement (AMPLE—Voyvodic, 2006). In this technique, *T*-values are scaled by a local (within ROI) maximum value just before thresholding. This results in reduced sensitivity to sample size and increased test-retest reliability (Voyvodic, 2012). However, this approach does not assume any formal model of noise and signal and does not incorporate spatial information, although this might not be necessary for small ROIs. It does, on the other hand, apply different thresholds for different parts of the brain. In principle it is very likely that characteristics of noise and signal are not stationary across the brain, but finding ROIs to fit models locally is not trivial. AMPLE uses ROIS that are atlas derived, manually drawn (Voyvodic, 2006; Voyvodic et al., 2009), or semi-automatically discovered from the same activation signal (Voyvodic, 2012). We aimed at keeping our method as automated as possible to reduce user input and subjectivity. Additionally using parcellation derived from activation signal to establish local parameters used for thresholding the same activation may introduce “double dipping” biases. Nonetheless, we can see a potential extension of the method in which mixture model could be fitted separately to different brain regions. In such approach parcellation and local thresholding should be done in an iterative way so one would inform the other until reaching convergence.

Because AT separates signal from noise, it was expected to reduce the false negative rate. Indeed, simulations show that AT has better Type II cluster error rates than FT, but this comes at the price of creating more false positive clusters. However, overall it achieved a better balance in terms of detection. One possible explanation for this is that AT tends to use lower cluster forming thresholds than the default FT values and thus good balance could be achieved simply by using a lower fixed threshold. Additional analyzes (see the Appendix) using two such low fixed thresholds, one corresponding to the mean threshold estimated with AT at high SNR and the other with AT at low SNR, show that this was not the case and that AT always outperforms FT because it adjusts to the estimated strength of the signal, thereby providing a lower threshold for weak signals and higher threshold for stronger signals. This results in fewer false negative clusters for weak signal cases and fewer false positives for strong signal cases. Despite the good balance obtained between false positive and negative clusters in our simulations, this method does not provide any guaranteed statistical properties. It is more of a heuristic approach based on sound assumptions than an analytical solution. A possible extension of the method that could improve sensibility is to fix the cluster forming threshold to a certain point, e.g., 0.05, on the cumulative density function of the signal distribution rather than using a point of equal probability between signal and noise. This would control explicitly for the expected voxel-wise Type II error rate. However, because this approach would not include information about the characteristic of noise, it will not be as accurate in terms of spatial extend and reliability.

Finally, because AT provides a higher spatial accuracy and adapts to noise, it also leads to an increase in reliability. In the context of single subject fMRI analysis, and in particular for data used in clinical procedures such as presurgical planning, it is worth noting that spatial accuracy is essential. Of particular interest here, AT showed much lower underestimation than FT, which may be useful in clinical situations. Increased spatial reliability in healthy controls also means that one can be confident that the method will more often detect valid clusters as suggested by the reduced false negative rate in the simulations. Overall, AT therefore achieves a better balance than FT approaches, and provides a new tool for reliably and objectively threshold multiple single-subject SPMs.

### Conflict of interest statement

The authors declare that the research was conducted in the absence of any commercial or financial relationships that could be construed as a potential conflict of interest.

## Figures and Tables

**Figure A1:**
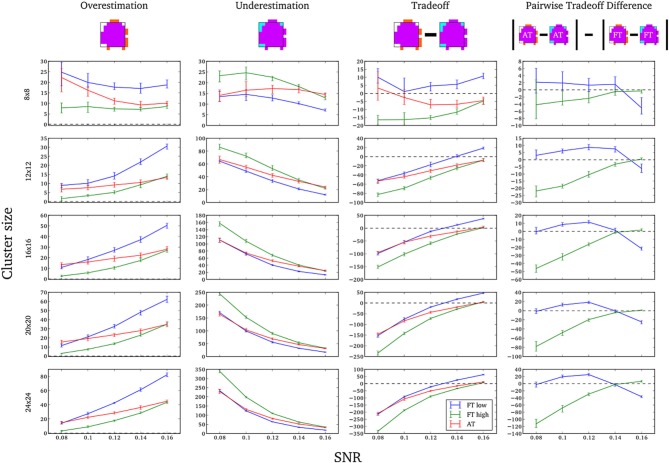
**Illustration of over and underestimation, and performances of AT and FT with low clusterforming thresholds**. At the top is an illustration of an observed cluster (purple) over the true underlying signal (square outline). Estimated voxels outside of the true border are in orange and missed voxels inside the border are in cyan. Below, graphs represent four biases. On the left are displayed the H-D estimates of the median cluster extent overestimation (number of false positive voxels for a particular cluster). Next are displayed the H-D estimates of the median cluster extent underestimation (number of false negative voxels for a particular cluster). Next is displayed the overestimation and underestimation trade-off (differences of the H-D estimates of the medians). Finally on the right hand side is displayed the pairwise comparison between AT and FT trade-offs. Each row corresponds to different cluster size and whiskers represent 95% confidence intervals. Due to the fact that the smallest cluster (4 × 4) was found by all of the thresholding methods only in a handful of runs it was excluded from this plot.

## Appendix

### Comparison of AT to FT using lower cluster forming thresholds

In this additional comparison we looked at two different fixed thresholds, specifically a low FT value was set to the mean threshold estimated by AT for low SNR cases and a high FT value was set to the mean threshold estimated by AT for high SNR cases. Pairwise trade-off difference showed that AT performs equally well as FT low and outperforms FT high for low SNR. This is reversed for high SNR. Therefore using the same fixed threshold (FT low or high) for any SNR situation leads to spatial inaccuracy of the border estimation which can be avoided by using AT (Figure A1).

**Table A1:** **Statistical analysis the pairwise difference (AT-FT) comparison of spatial accuracy trade-off (see last column of Figure 3)**.

			**SNR**									
			**0.08**		**0.1**		**0.12**		**0.14**		**0.16**	
			**0.001**	**0.05 FWE**	**0.001**	**0.05 FWE**	**0.001**	**0.05 FWE**	**0.001**	**0.05 FWE**	**0.001**	**0.05 FWE**
Cluster size	8 × 8	High CI	17.624	−17.949	15.924	−24.583	−0.985	−30.220	0.842	−26.872	0.084	−25.104
		H-D median	−10.574	−35.951	0.209	−36.159	−4.722	−33.886	−0.208	−28.597	−0.323	−26.397
		Low CI	−19.000	−41.000	−8.616	−38.967	−7.310	−36.614	−1.961	−30.305	−1.244	−27.710
		q-value	0.720	**0.000**	0.985	**0.000**	**0.031**	**0.000**	0.824	**0.000**	0.179	**0.000**
	12 × 12	High CI	−21.769	−67.618	−22.629	−70.496	−18.924	−71.547	−10.301	−62.223	−2.035	−51.868
		H-D median	−26.068	−76.810	−25.523	−76.621	−20.370	−74.153	−12.286	−64.090	−3.455	−54.108
		Low CI	−29.245	−79.166	−27.671	−79.760	−22.375	−75.860	−14.309	−65.932	−4.912	−55.846
		q-value	**0.000**	**0.000**	**0.000**	**0.000**	**0.000**	**0.000**	**0.000**	**0.000**	**0.000**	**0.000**
	16 × 16	High CI	−54.657	−137.005	−40.853	−146.946	−29.412	−122.378	−11.873	−93.170	1.494	−65.270
		H-D median	−62.178	−143.510	−45.686	−152.128	−33.017	−126.985	−14.093	−97.528	0.032	−67.542
		Low CI	−67.046	−153.732	−49.917	−156.135	−36.206	−132.445	−16.313	−101.854	−1.874	−70.395
		q-value	**0.000**	**0.000**	**0.000**	**0.000**	**0.000**	**0.000**	**0.000**	**0.000**	0.976	**0.000**
	20 × 20	High CI	−72.921	−204.005	−69.943	−234.604	−40.932	−192.090	−17.569	−130.588	−0.399	−85.261
		H-D median	−89.445	−218.161	−76.181	−241.916	−44.158	−196.763	−20.186	−137.650	−3.039	−89.292
		Low CI	−101.879	−228.995	−83.028	−247.105	−46.580	−202.319	−22.103	−144.764	−6.096	−95.166
		q-value	**0.000**	**0.000**	**0.000**	**0.000**	**0.000**	**0.000**	**0.000**	**0.000**	**0.035**	**0.000**
	24 × 24	High CI	−136.983	−311.630	−109.479	−344.577	−58.295	−280.437	−20.326	−185.564	3.943	−111.384
		H-D median	−150.097	−324.914	−120.186	−356.117	−60.786	−287.782	−24.314	−188.970	1.566	−117.165
		Low CI	−163.942	−336.446	−129.655	−365.877	−64.013	−293.151	−27.205	−192.819	−0.169	−121.351
		q-value	**0.000**	**0.000**	**0.000**	**0.000**	**0.000**	**0.000**	**0.000**	**0.000**	0.094	**0.000**

Q-Values correspond to p-values that were corrected for multiple comparisons using B-H method for controling FDR.

Statistically significant tests are highlighted in bold.
